# Fueling AI literacy through school support: unveiling the mediating role of basic psychological need satisfaction in Chinese university English teachers

**DOI:** 10.1186/s40359-025-03949-6

**Published:** 2026-01-07

**Authors:** Jie Yang, Xiangling Li

**Affiliations:** School of International Education, Henan University, Kaifeng, China

**Keywords:** AI literacy, School support, Self-Determination theory, Basic psychological needs, University English teaching

## Abstract

Although the growing importance of AI integration in education is widely acknowledged, limited empirical research has explored how school support addressing teachers’ basic psychological needs fosters AI literacy, especially in Chinese higher education. Grounded in Self-Determination Theory (SDT), this study investigated how school support influences the development of AI literacy among university English teachers in China, focusing on the roles of autonomy, competence, and relatedness. In this study, AI literacy involves understanding, application, evaluation, and ethical reflection on AI technology—key factors in educational technology integration. Through survey data from 412 teachers and semi-structured interviews with 23 teachers, the study developed and validated a model linking school support, basic psychological need satisfaction (BPNS), and AI literacy. Quantitative analysis indicated that school support enhances teachers’ BPNS, which in turn mediates the impact of school support on AI literacy. Qualitative findings identified several key institutional strategies that address teachers’ autonomy, competence, and relatedness, promoting AI literacy development. Together, these findings highlight the pivotal role of school support and teachers’ BPNS in fostering AI literacy, providing insights for educational policy and teacher professional development.

**Supplementary Information:**

The online version contains supplementary material available at 10.1186/s40359-025-03949-6.

## Introduction

The swift advancement of artificial intelligence (AI) technology globally has significantly propelled the digital transformation of education, prompting educators to reassess their teaching methodologies. AI technology not only offers personalized learning experiences for students and alleviates teachers’ workloads (such as grading and performance analysis) but also enhances teaching efficiency through precise, continuous feedback, ultimately promoting educational equity [1–4]. This transformation is particularly evident in the field of language teaching, where AI technologies, from intelligent teaching tools to smart learning platforms, are reshaping the modalities and methods of language acquisition [5–8].

To fully harness the potential of AI technology, teachers must possess a strong foundation in AI literacy. AI literacy encompasses a deep understanding of fundamental AI principles, effective use of AI tools, critical evaluation of their applications, and the ability to innovate within frameworks of ethical and social responsibility [9, 10]. In China, university English teachers represent a key educational group. They are increasingly expected to integrate AI into their teaching and to guide students in understanding AI-related ethical issues and responsibilities, thereby promoting students’ critical thinking and innovation skills [10, 11].

While the importance of AI literacy has been widely acknowledged, most existing research predominantly emphasizes skill-based training, with limited attention paid to how addressing teachers’ basic psychological need satisfaction (BPNS) can support their acceptance and use of AI technology. According to Self-Determination Theory, satisfying teachers’ basic psychological needs—autonomy, competence, and relatedness—is essential for promoting motivation, capacity development, and professional growth [12–14]. When these psychological needs are fulfilled, teachers are more likely to embrace new technologies, engage in professional development, and improve both teaching effectiveness and job satisfaction [15, 16]. However, empirical studies examining how institutional support can help fulfill these psychological needs to advance AI literacy, particularly in the context of Chinese higher education, remain scarce.

Meanwhile, China’s government has introduced several policy initiatives to promote AI integration in education, such as *China Education Modernization 2035* [17] and *The Action Plan for Artificial Intelligence Innovation in Higher Education Institutions* [18]. These policies also call for improving teachers’ AI literacy to support educational digitalization. However, specific policy support targeting the unique needs of university English teachers remains limited, despite this growing attention. In the broader Chinese EFL context, the number of English learners was estimated at over 390 million in 2000 [19] and has continued to grow [20], underscoring the scale and ongoing expansion of English education in China. With approximately 40 million university English learners in China [21], educational resources are under significant strain. While AI technology presents potential solutions to alleviate this pressure, teachers’ gaps in AI training and application skills remain major obstacles to effective AI integration [22–24]. Research indicates that many Chinese university English teachers lack sufficient AI literacy, displaying deficits in both knowledge and skills, and often lack confidence in employing AI in the classroom [25, 26]. Additionally, unmet needs for autonomy, competence, and relatedness in AI technology use further hinder effective integration [15, 27].

In response to the challenges educators face in implementing digital technologies, multiple frameworks have been developed, including the European Framework for the Digital Competence of Educators (DigCompEdu), the ISTE Standards for Educators, the Digital Literacy Standards for Teachers in China, and more recently, UNESCO’s AI Competency Framework for Teachers [28–31]. Although these frameworks provide structured guidance for enhancing AI literacy skills, they do not specifically address the needs of university English teachers in China.

Therefore, this study aimed to address this gap through a mixed-methods approach, examining how institutional support influences Chinese university English teachers’ BPNS and fosters their development of AI literacy. The findings offer practical insights for educational administration and elucidate the mechanisms that connect teachers’ BPNS with the development of AI literacy in the context of Chinese higher education.

## Literature review

### Teachers’ AI literacy

AI encompasses computer systems designed to mimic human intelligence, assisting users in a variety of tasks and problem-solving [32]. Recent breakthroughs in deep learning, neural network algorithms, and computational power have accelerated AI’s application across multiple sectors [33, 34]. In education, AI has been integrated into various contexts, enabling teachers to explore intelligent teaching models, enhance personalized learning experiences, facilitate evidence-based research, and support data-driven decision-making in educational administration [35–38]. However, the high technical demands and interdisciplinary nature of AI in education present significant challenges for developing and implementing effective learning activities [39]. Consequently, the importance of teachers’ AI literacy has become increasingly pronounced.

The term “AI literacy” was first introduced by Konishi [40] and includes not only the understanding and application of AI technologies but also a deeper cognition and critical thinking toward AI. Although definitions of teachers’ AI literacy vary, a consensus emerges that it extends beyond traditional information, data, and digital literacy, reflecting an evolution of these competencies for the intelligent era [41, 42]. Teachers’ AI literacy is thus viewed as a multifaceted skill set that integrates knowledge, skills, attitudes, and ethics, enabling educators to effectively navigate AI-driven teaching environments [10, 43]. Recent research has proposed several frameworks to systematically analyze and construct the components of AI literacy. For example, Touretzky et al. introduced a framework consisting of five key AI concepts: perception, representation, reasoning, learning, and natural interaction, aimed at elucidating fundamental AI principles in education [44]; Cetindamar et al. identified four core competencies of AI literacy—technology, work, human-machine interaction, and learning—highlighting AI’s extensive applicability in professional settings [45]. While these theoretical frameworks offer valuable insights, their practical application in educational settings poses challenges. In China, national policy initiatives and the accelerated pace of educational informatization have further underscored the need to enhance teachers’ AI literacy, particularly in higher education. Although research in Computer-Assisted Language Learning (CALL) has established the importance of information technology in language instruction [46, 47], studies reveal that many university English teachers struggle to effectively integrate AI technologies into their teaching [48]. The absence of clear application strategies often leads to superficial technology use, preventing the realization of its full educational potential [49]. Additionally, teachers frequently encounter difficulties in bridging theoretical concepts with practical classroom needs, further complicating the effective application of AI technologies [50–52]. As a result, enhancing teachers’ AI literacy has become a pressing priority.

To address these challenges, this study employed the four-dimensional AI literacy framework developed by Ng et al. [53]. Tailored for teachers, this framework comprises understanding AI, applying AI, evaluating and creating AI, and considering AI ethics. It provides robust theoretical guidance for teaching practices in the digital age while tackling practical issues related to enhancing AI literacy in foreign language instruction. Drawing on Bloom’s taxonomy of educational objectives, it spans various cognitive levels, from foundational understanding to advanced application. By integrating this framework, the study aimed to equip teachers to meet the demands of AI technology, thereby providing theoretical support for their AI literacy and facilitating the incorporation of AI technologies to enhance teaching.

### The role of teachers’ BPNS in AI literacy

Self-Determination Theory (SDT) serves as a pivotal theoretical framework for understanding teacher motivation, with far-reaching implications for both traditional and technology-enhanced teaching practices [13, 14]. SDT posits that individuals must satisfy three basic psychological needs—autonomy, competence, and relatedness—throughout their lives to achieve optimal functioning, personal growth, and well-being. When these needs are met, intrinsic motivation is fostered, leading to sustained engagement. Conversely, when these needs are unmet, individuals often rely on external motivators (such as rewards or punishments), which may facilitate task completion but do not support long-term motivation.

SDT has been widely applied in educational research, with a primary focus on student motivation and engagement. Studies have shown that satisfying students’ basic psychological needs positively influences their engagement and academic achievement [54–56]. Similarly, for teachers, fulfilling these basic psychological needs can enhance their teaching motivation, thereby facilitating the integration of new technologies in their instructional practices [1, 57].

Importantly, beyond motivation, BPNS may also play a critical role in the development of teachers’ AI literacy. When teachers feel autonomous, competent, and connected in their professional environment, they are more likely to value the integration of AI and engage willingly in related learning and teaching practices. As AI literacy encompasses understanding, application, evaluation, and ethical reflection on AI technologies (as discussed in Sect. 2.1), such intrinsic motivation fosters deeper and sustained engagement in acquiring and applying AI knowledge and skills. In this way, BPNS functions not only as a motivational factor but as a psychological foundation that supports teachers’ meaningful and long-term investment in AI literacy development.

However, existing research has largely focused on the direct impact of teacher motivation on their instructional behavior, with limited exploration of how external support—such as that from schools—can assist teachers in meeting these basic psychological needs to improve teaching practices [12]. Particularly within the context of teachers’ ongoing learning and motivation regarding AI technology use, the literature reveals a notable deficiency in understanding how schools and policymakers can implement supportive measures to enhance teachers’ technological practices [15, 58]. This research gap aligns with the suggestions of SDT’s founders to further investigate the role of schools in motivating teachers [13, 14].

Consequently, this study aimed to fill this gap by examining teachers’ BPNS and the role this satisfaction plays in the development of AI literacy, thereby providing both theoretical foundations and empirical evidence on how schools can better support teachers in adopting AI technologies.

### School support and teachers’ AI literacy

School support generally refers to the resources, environment, and assistance provided by educational institutions to promote the academic and personal growth of both students and teachers [59]. From the perspective of SDT, when school support aligns with teachers’ basic psychological needs, they are more inclined to connect with the school’s objectives and embrace new instructional challenges [60, 61]. This is because satisfying needs for autonomy, competence, and relatedness nurtures teachers’ intrinsic motivation [62]. Evidence from SDT research suggests that a supportive school environment—characterized by a positive culture, effective leadership, and collegial collaboration—can substantially enhance teachers’ motivation, engagement, and professional commitment [12, 63, 64]. In the context of technology integration, the extent of school support directly affects teachers’ BPNS and their intrinsic motivation, which subsequently influences their use of technology [15, 65, 66]. Given the multidimensional nature of AI literacy, school support that effectively addresses teachers’ psychological needs may create favorable conditions for them to acquire, apply, and reflect on AI knowledge and skills, thereby advancing their AI literacy development.

Despite the focus of current research on teachers’ application of AI technologies in the classroom, there remains a significant need to explore how schools can systematically improve teachers’ AI literacy through targeted support, policy initiatives, and resource allocation. Specifically, the literature lacks comprehensive studies on how school support strategies can facilitate teachers’ integration of AI technologies in teaching practices. This study aimed to explore how school support influences English teachers’ BPNS, which in turn enhances their AI literacy. Through this, the study intends to contribute to the development of support strategies in educational settings that can foster both teacher well-being and the effective integration of AI technologies.

## Methods

### Research questions and hypotheses

Teachers’ AI literacy is a key competency for effectively integrating technology into education. This study adopted Ng’s AI literacy framework [10, 53], grounded in Bloom’s taxonomy of cognitive learning [67], which progresses from foundational understanding to practical application, evaluation, and ethical reflection on AI.

**Fig. 1:**
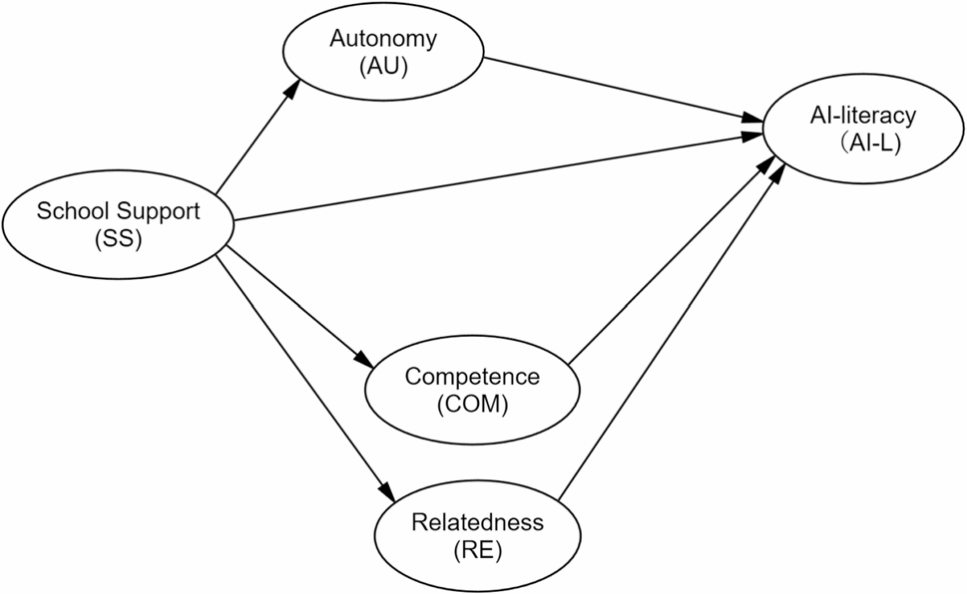
Research model

Developing teachers’ AI literacy is influenced not only by self-directed learning but also by institutional support. According to SDT, satisfying teachers’ basic psychological needs is crucial for enhancing their motivation and performance [62]. Understanding how school support interacts with these needs can provide insight into how institutions foster AI literacy among educators. Building on this theoretical foundation, this study examined the potential mediating role of teachers’ BPNS in the relationship between perceived school support and teachers’ AI literacy (see Fig. 1). The following research questions and hypotheses are framed to explore these dynamics:

- RQ1: Do university English teachers’ BPNS (autonomy, competence, relatedness) mediate the relationship between perceived school support and their AI literacy?
- RQ2: What types of school support would satisfy university English teachers’ BPNS, thereby enhancing their AI literacy and improving their integration of AI into teaching?

To address RQ1, the study proposes the following hypotheses:

- H1: School support positively influences teachers’ BPNS.
- H2: The satisfaction of each basic psychological need positively influences teachers’ AI literacy.
- H3: Teachers’ BPNS mediates the relationship between school support and teachers’ AI literacy.

### Participants

This study involved university English teachers from various higher education institutions in China. A total of 412 educators participated in the survey, representing a diverse range of institutions, including first-tier, second-tier, third-tier, and vocational colleges. To gain a thorough understanding of the current state of teachers’ AI literacy, the survey collected demographic information, including gender, years of teaching experience, educational background, and type of institution. A detailed demographic profile of the participants is presented in Table 1.

**Table 1:** The participants’ demographic profile (*N* = 412)

Measure	Category	Amount	Percentage
Gender	Male	93	22.6
	Female	319	77.4
Years of experience as teacher	< 5	94	22.8
	6–10	68	16.5
	11–15	74	17.9
	16–20	69	16.8
	21–25 > 25	72 35	17.5 8.5
Educational credentials	Bachelor’s	50	12.1
	Master’s	295	71.6
	Doctorate	67	16.3
University type	First-tier universities^a^	232	56.3
	Second-tier universities^b^	119	28.9
	Third-tier universities^c^	37	9.0
	Vocational colleges^d^	24	5.8

^a^: Top universities; ^b^: Mid-tier universities; ^c^: Lower-tier or private universities; ^d^: Colleges focus on practical skills

In addition, to explore RQ2, the study conducted semi-structured interviews with 23 teachers selected through purposive sampling to ensure diverse representation across various institutions as well as variation in teaching experience and administrative roles, thereby capturing a broad range of perspectives relevant to teachers’ AI literacy development. The sample comprised 20 frontline teachers (five of whom were department heads with combined teaching and leadership roles) and three administrators (a vice principal, a dean, and a head of the information management center), each contributing a range of teaching experiences and backgrounds. The qualitative data from these interviews provided a substantial foundation for analysis, enriching the survey results. The detailed demographic characteristics of the interview participants are provided in Appendix B.

### Research instruments

This study employed two primary research instruments: a questionnaire survey and semi-structured interviews, tailored to address RQ1 and RQ2, respectively.

#### Questionnaire survey

The questionnaire was developed based on established theoretical frameworks and covered the following key dimensions:

- *School support*: Adapted from Lee et al. [61], this dimension consists of four items assessing teachers' perceptions of school support, yielding a Cronbach's α coefficient of 0.854.
- *BPNS* (autonomy, competence, relatedness): Based on Chen et al. and Chiu et al. [12, 68], this dimension comprises three sub-dimensions—autonomy, competence, and relatedness—each containing four items. The Cronbach’s α coefficients for these sub-dimensions are 0.892, 0.898, and 0.884, respectively.
- *AI literacy* (know and understand AI, use and apply AI, evaluate and create AI, AI ethics): Drawing from Ng et al. and Carolus et al. [10, 11], this dimension includes four sub-dimensions—knowledge, application, evaluation, and ethics—each with five items. All sub-dimensions demonstrate Cronbach’s α coefficients above 0.86 (0.903, 0.868, 0.864, 0.890).

The questionnaire utilized a 5-point Likert scale (1 = strongly disagree, 5 = strongly agree), exhibiting acceptable reliability and validity. In addition to the reliability indicators mentioned above, content validity was ensured by adopting an established theoretical framework. Construct validity was assessed using both exploratory factor analysis (EFA) and confirmatory factor analysis (CFA). EFA results indicated satisfactory factor structure (KMO = 0.938; Bartlett’s Test *p* < 0.001; cumulative variance explained = 71.63%). CFA demonstrated good model fit (*χ²*/*df* = 1.184; GFI = 0.917; CFI = 0.988; NFI = 0.927; IFI = 0.988; RMSEA = 0.021). All standardized factor loadings exceeded 0.5 and were statistically significant (*p* < 0.001). Convergent validity was supported (CR values > 0.856; AVE values > 0.566), and discriminant validity was supported, as the square roots of AVE surpassed inter-construct correlations.

The complete questionnaire is provided in Appendix A.

#### Semi-structured interviews

To further explore the impact of school support on teachers’ AI literacy, the study developed interview questions anchored in SDT, drawing from the work of Chiu and Chiu et al. [12, 15]. Semi-structured interviews were conducted with the 23 participants, focusing on the following key areas: support for teachers’ autonomy in selecting AI tools; assistance in developing their AI-related competencies and confidence; and facilitation of collaboration and communication regarding AI technologies among teachers. The full interview protocol can be found in Appendix C.

### Data collection and analysis

#### Data collection

This study received approval from the Ethics Committee of the affiliated institution and strictly adhered to ethical guidelines. In the first phase, the questionnaire was distributed online via platforms such as Wenjuanxing to English teachers at universities across China. A total of 436 teachers participated; after excluding responses with inconsistencies or those completed in under the minimum time, 412 valid responses remained, resulting in an effective response rate of 94.5%. In the second phase, interview participants were selected from those who voluntarily provided their contact information in the questionnaire. Each interview lasted approximately 25 min. Four participants declined to consent to audio recording, so their responses were captured through detailed notes. For those interviews where recording was permitted, the audio was transcribed and organized, forming the basis for subsequent qualitative data analysis.

#### Data analysis

This study utilized a mixed-methods approach, combining quantitative and qualitative analyses to address the two research questions.

To answer RQ1, confirmatory factor analysis (CFA) was conducted to evaluate the measurement model’s quality and examine relationships among the latent variables in the questionnaire. Subsequently, structural equation modeling (SEM) was carried out using IBM SPSS Amos 26 version. The analysis was based on maximum likelihood estimation, and 5000 bootstrap resamples were drawn to assess the indirect effects within the model [69, 70].

To address RQ2, SDT was used as the analytical framework, and a deductive content analysis approach was applied to the interview data [71]. This analysis explored how school support enhances teachers’ AI literacy by fulfilling their intrinsic needs. The analysis proceeded in three phases: (1) Preliminary categorization: Two raters categorized teacher responses based on SDT; (2) Identification and refinement: Coding extracted strategies supporting teachers’ needs, which were then refined into subcategories; (3) Optimization of classification: Subcategories were further refined to clarify how school support promotes the development of teachers’ AI literacy.

To ensure the validity and reliability of the analysis, the following strategies were implemented:

- Appropriateness: Interview questions were designed based on relevant studies by Chiu and Chiu et al. to align with the study’s context [12, 15].
- Triangulation: Multiple data sources, including policy documents and teaching materials provided by teachers, were incorporated to enhance the comprehensiveness of the findings.
- Credibility: Interviews were conducted by two experienced researchers. The first author, a frontline teacher with 10 years of university English teaching experience, and the corresponding author, a former associate dean and current dean of the School of Foreign Languages, provided valuable insights into teachers’ needs and school support dynamics. To mitigate potential bias, inter-rater calibration and participant feedback were employed to ensure the professionalism and rigor of the findings.
- Ethical compliance: The study received approval from the Ethics Committee, with data collection respecting participants’ rights.

## Research results

### Results of quantitative analysis: the mediating role of teachers’ BPNS

To address RQ1, SEM was used to examine the relationships among variables in the hypothesized model. School support was designated as the predictor variable, with university English teachers’ BPNS (autonomy, competence, and relatedness) serving as mediating variables, and teachers’ AI literacy as the criterion variable.

#### Results of descriptive analysis

Table 2 presents the descriptive statistics for each variable. The skewness and kurtosis values indicate minor deviations from normality; however, these remain within the acceptable range for standard statistical analyses. To enhance the robustness of the findings, the bootstrap method was applied in subsequent analyses. The results of the correlation analysis (see Table 3) show significant positive correlations among all variables (*p* < 0.01), establishing a foundation for the subsequent path analysis and mediation effect tests. Notably, when judged against Wei et al.‘s benchmarks [72], the observed coefficients (ranging from 0.304 to 0.516) fall within the range of large effect sizes, underscoring their practical relevance.

**Table 2:** Descriptive statistics for the questionnaire

Measure	Mean		SD	Skewness	Kurtosis
School support	3.41		0.98	−0.27	−0.21
Autonomy	3.81		0.95	−0.63	0.09
Competence	3.26		1.10	−0.04	−0.86
Relatedness	3.53		1.06	−0.36	−0.51
AI-literacy	3.66		0.72	−0.08	−0.50

**Table 3:** Correlation among all the variables in research model

Variables	1	2	3	4	5
1. School support	-				
2. Autonomy	0.325**	-			
3.Competence	0.304**	0.343**	-		
4. Relatedness	0.329**	0.406**	0.384**	-	
5. AI-literacy	0.405**	0.516**	0.423**	0.471**	-

**p < 0.01

#### Results of confirmatory factor analysis and structural equation modeling

The result of CFA demonstrates strong convergent validity and reliability for the measurement model. All factor loadings exceed 0.70, and Cronbach’s α values are above 0.85, reflecting excellent internal consistency across scales. Additionally, the fit indices confirm the model’s suitability: *χ²*/*df* = 1.184 (< 3.0), RMSEA = 0.021 (< 0.08); GFI = 0.917 (> 0.90); CFI = 0.988 (> 0.90); NFI = 0.927 (> 0.90); IFI = 0.988 (> 0.90) [73].

**Fig. 2:**
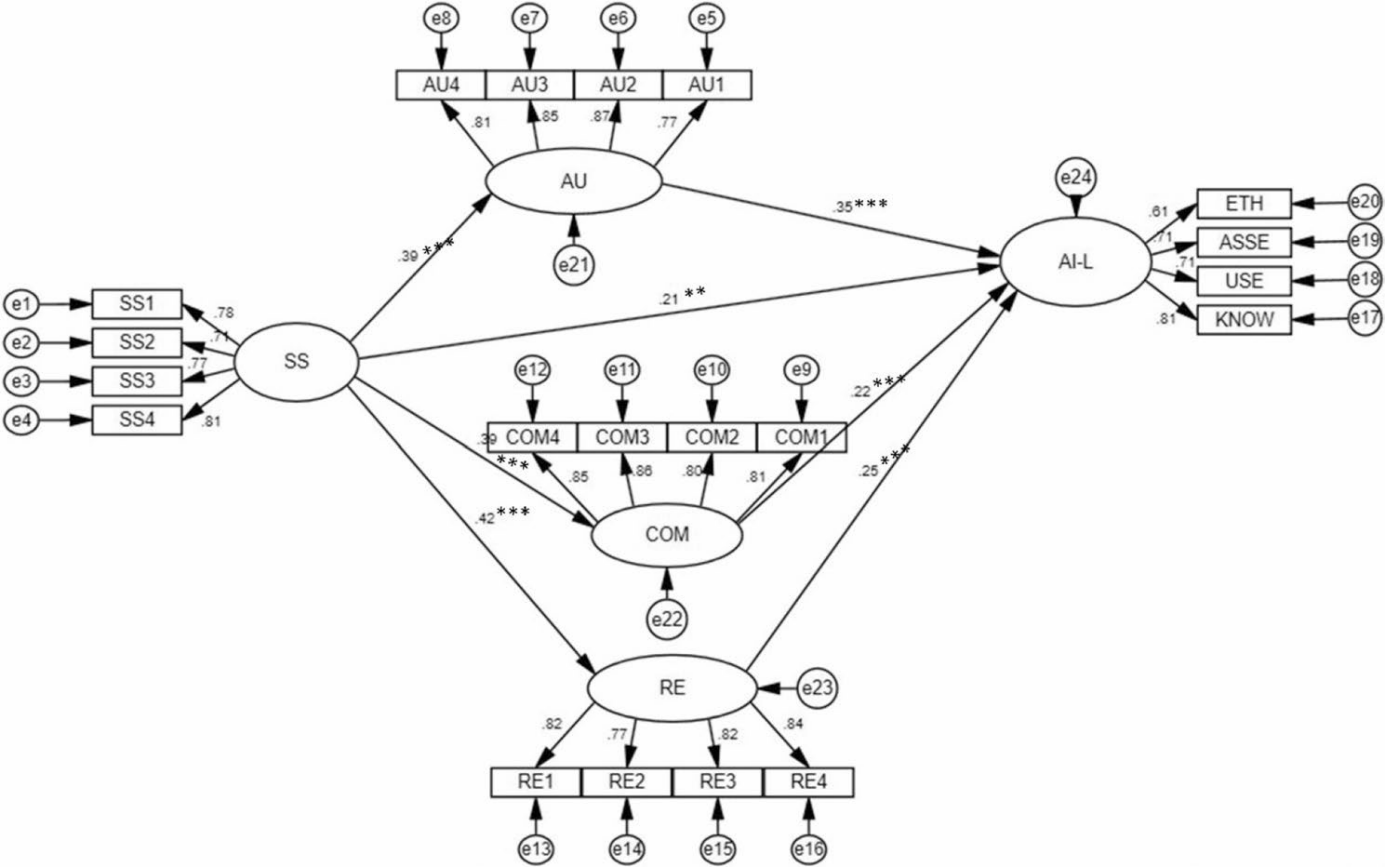
Final Structural Equation Model. ***: p < 0.001; **: p < 0.01; SS = School support; AU = autonomy; COM = Competence; RE = Relatedness; AI-L = AI literacy

Further SEM analysis indicates a good fit between the research model and the data, with fit indices as follows: *χ²*/*df* = 1.735 (< 3.0), RMSEA = 0.042 (< 0.08), GFI = 0.917 (> 0.90), CFI = 0.973 (> 0.90), NFI = 0.937 (> 0.90), and IFI = 0.973 (> 0.90). These values indicate an excellent model-data fit. Figure 2 illustrates the path relationships and coefficients among variables in the model, while Table 4 provides detailed results of standardized direct and indirect effects.

**Table 4:** Standardized direct, indirect effects among the variables in the research model

Path relationships	Direct effect (SS–> AI-L)	Indirect effect	Bias-corrected (95%)		*p*	Conclusion
			Lower Bounds	Upper Bounds		
SS AU AI-L	0.209	0.112	0.058	0.189	0.001	partial mediation
SS COM AI-L	0.209	0.070	0.029	0.133	0.001	partial mediation
SS RE AI-L	0.209	0.087	0.037	0.162	0.001	partial mediation

The statistical results supported all research hypotheses:

- H1: School support positively influences teachers’ BPNS. Perceived school support significantly influences the satisfaction of teachers’ needs for autonomy (*β* = 0.39, *p* < 0.001), competence (*β* = 0.389, *p* < 0.001), and relatedness (*β* = 0.42, *p* < 0.001).
- H2: The satisfaction of each basic psychological need positively influences teachers’ AI literacy. Results indicate that satisfying teachers’ needs for autonomy (*β* = 0.352, *p* < 0.001), competence (*β* = 0.221, *p* < 0.001), and relatedness (*β* = 0.252, *p* < 0.001) significantly enhances their AI literacy.
- H3: Teachers’ BPNS mediates the relationship between school support and teachers’ AI literacy. Mediation analysis results indicate that teachers’ BPNS plays a significant mediating role between school support and AI literacy. School support not only directly impacts AI literacy positively (*β* = 0.209, *p* = 0.001) but also exerts an indirect influence through teachers’ BPNS (see Table 4).

These indirect effects were verified using the bootstrap method, with a 95% confidence interval that excludes zero, indicating significant mediation effects [73, 74].

The results show an R² of 0.488 for AI literacy, representing a large effect under Wei et al.‘s benchmarks [72]. These benchmarks, also adopted in recent survey-based studies [75–77], support the broader applicability of this interpretation. This relatively high effect is reasonable, as school support and teachers’ basic psychological needs are conceptually and empirically tied to professional competence and technology use. The findings indicate that school support, autonomy, competence, and relatedness together explain a meaningful share of variance in teachers’ AI literacy, reflecting both statistical significance and practical value.

### Results of qualitative analysis: school support strategies for AI literacy

To address RQ2, this study adopted an analytical framework grounded in SDT and applied deductive content analysis to examine interview data, relevant policies, and teaching materials. To ensure confidentiality, participants are identified by unique codes (e.g., P01, P02 for teachers; A01, A02 for administrators). Detailed participant information is provided in Appendix B. The analysis identified three key areas of support strategies: autonomy, competence, and relatedness.

#### Autonomy support strategy: creating flexible learning spaces and opportunities for exploration

Many teachers pointed out that their initial exposure to AI technology came primarily through external training programs or recommendations of colleagues. However, they emphasized that genuine skill improvement happens when the school allowes them to explore AI applications in their own space and according to their teaching needs.

“After the training, our department encouraged us to try different AI tools in our own classes. I could decide how to use them, and that really made me feel more confident and creative in my teaching.“(P02).

“What I like most is that there isn’t a fixed format. We’re free to choose the platforms or tools that work best for us.“(P05).

Nonetheless, some teachers mentioned that the cost of AI tools limited their ability to explore further:

“I’d love to try out some of the advanced AI tools, but many of them charge fees, and the school doesn’t really cover those costs.” (P15).

“If the school could offer more funding or shared licenses for AI platforms, I think a lot more teachers would be willing to experiment and explore on their own.” (P16).

Overall, teachers desire learning paths initiated by the school yet controlled by themselves. Schools can support this by providing flexible learning platforms, dedicated time for experimentation, and sufficient resources that enable teachers to select learning content independently. Financial support and other enabling measures can further reduce external barriers, strengthening teachers’ intrinsic motivation and enhancing their AI literacy.

#### Competence support strategy: strengthening technical mastery and ongoing training mechanisms

Teachers generally hold that improving AI application skills requires ongoing and context-specific training rather than one-off technical demonstrations.

“We need AI training that can be directly applied to subject teaching.“(P18).

“Only through practice can we truly master AI applications.“(P13).

Beyond formal training, many teachers also emphasized the importance of continuous technical support. While short-term workshops may spark interest, they are insufficient for addressing the detailed issues encountered in a real classroom context.

“What we need is continuous support, not just one or two training sessions.“(P10).

“The school should regularly help us evaluate how AI actually improves our teaching.” (P19).

Ongoing technical support and feedback are essential for building teachers’ confidence and improving their practical skills. By providing sustained and context-relevant support, schools can strengthen teachers’ professional confidence and promote the effective integration of AI into teaching practice.

#### Relatedness support strategy: constructing social support and collaborative networks

Teachers consistently highlighted the unique importance of learning communities and peer interactions in AI teaching:

“In the learning community, interacting with other teachers has taught me a lot.“(P01).

By engaging in community discussions, sharing case studies, and collaborating on projects, teachers find both emotional support and practical ideas that inspire their AI teaching:

“The community gave me the opportunity to learn from my colleagues and grow together.“(A02).

These peer interactions not only help reduce the anxiety teachers feel when integrating new technologies but also foster stronger connections and a deeper sense of belonging. Several teachers mentioned that guidance from more experienced peers greatly accelerated their adaptation to AI:

“With the help of seasoned teachers, I was able to better grasp AI technology.” (P01).

Additionally, teachers stress the value of interdisciplinary collaboration. One teacher shared:

“We collaborated with IT teachers to develop teaching materials, and it worked really well.” (P07).

Therefore, schools should promote open communication and interdisciplinary collaboration, providing mentoring and community support. This will strengthen teachers’ professional growth and ultimately enhance their AI literacy.

By addressing the basic psychological needs of autonomy, competence, and relatedness, school support strategies have contributed to enhancing teachers’ AI literacy. Autonomy support motivates teachers through flexible learning platforms; competence support boosts their confidence with continuous training and feedback; and relatedness support strengthens emotional and professional recognition through peer and interdisciplinary collaboration. These strategies provide essential assistance for teachers’ successful integration of AI into their practice.

## Discussion

This study explored the impact of school support on university English teachers’ AI literacy. All proposed hypotheses were confirmed: school support positively influences teachers’ BPNS; satisfying these needs enhances teachers’ AI literacy; and BPNS mediates the relationship between school support and AI literacy. The qualitative analysis also identified several key school support strategies that effectively meet teachers’ BPNS, thereby facilitating AI literacy development. The following discussion relates these findings to previous research, placing them in a broader academic context.

### School support and teachers’ BPNS

The first hypothesis (H1) proposed that school support positively influences teachers’ BPNS, aligning with the principles of SDT. As emphasized by Ryan and Deci [13, 14], satisfying these needs is fundamental for individual motivation and well-being. This study affirmed that by providing technological resources, professional training, and collaborative opportunities, schools can effectively meet teachers’ psychological needs, increasing their motivation and readiness to incorporate AI technology into their teaching practices. Teachers often face stress and uncertainty with new technologies [78, 79]; however, school-provided training and technical support significantly enhance teachers’ operational skills and confidence, encouraging a proactive approach to AI adoption in education. This corroborated earlier findings, showing the significance of school support in facilitating teachers’ integration of new technologies [80, 81].

Further, granting teachers autonomy in course design and teaching methods has been shown to foster creativity and intrinsic motivation [82, 83]. Echoing this, our study found that when schools support teachers’ autonomy, they are more likely to creatively incorporate AI tools into their classrooms [84, 85]. Additionally, school-led peer collaboration initiatives strengthen teachers’ sense of belonging, a crucial factor for successful technology adoption. Although many teachers initially hesitate to use unfamiliar technology, collaboration within teacher communities helps ease these concerns and encourages a collective commitment to digital integration [86, 87].

School support bolstered teachers’ professional engagement while encouraging innovation and change-oriented behaviors [88]. In an era marked by AI advancements, such support enables teachers to embrace new technologies confidently, promoting both professional commitment and instructional innovation. These findings echoed previous studies on the contribution of leadership and resource support for teachers’ needs satisfaction [61, 89]. Taken together, these results indicate that meeting teachers’ needs for autonomy, competence, and relatedness is central to improving teaching effectiveness in the context of digital transformation.

### The role of school support in enhancing teachers’ AI literacy

The study identified a direct relationship between school support and teachers’ AI literacy. According to the four-dimensional AI literacy framework proposed by Ng et al. [10], teachers’ AI literacy includes not only technological understanding and application but also ethical awareness and critical evaluation skills. Our findings suggested that through resource allocation, targeted training, and technical support, schools act as key facilitators in nurturing these aspects of AI literacy. This aligned with prior research showing that school support promotes innovative teaching practices and that a supportive school culture and professional development programs enhance teachers’ ability to integrate technology [90–92], underscoring the importance of school working conditions (such as resources, environment, and institutional culture) in advancing teachers’ AI literacy.

Teacher AI literacy is a key factor in responding to the digital transformation of education, improving teaching efficiency, and promoting educational equity [93, 94]. Teachers with low AI literacy may face challenges in effectively incorporating AI technologies, which can negatively impact student outcomes. In contrast, teachers with high AI literacy can flexibly utilize AI tools to optimize decision-making, improve efficiency, and provide tailored learning support, meeting diverse student needs and maximizing student potential [10, 39]. Therefore, if schools implement diverse incentive strategies to enhance AI literacy, teachers will be better prepared to meet the demands of AI integration and achieve sustained professional growth. Similar to the findings of Chiu et al. [12], our study further validated the empirical link between teachers’ BPNS and technology adoption, highlighting a previously underexplored connection between school support and the enhancement of teachers’ AI literacy.

### The role of teachers’ BPNS as a mediator

The third hypothesis examined whether teachers’ BPNS mediates the relationship between school support and teachers’ AI literacy. Findings showed that school support directly promotes AI literacy, while also indirectly enhancing teachers’ technological skills by helping fulfill their needs for autonomy, competence, and relatedness. Specifically, autonomy was supported when teachers had more freedom and creativity in using AI tools; competence was strengthened through professional development and technical support that boosted teachers’ confidence in applying AI; and relatedness was encouraged through collaborative activities that fostered peer connection and a sense of belonging. Together, these three needs acted as a psychological pathway that translated school-level support into active teacher engagement with AI technologies. Meeting these needs facilitates AI adoption while encouraging instructional innovation and reflective practice.

Although SDT has extensively explored the connection between basic psychological needs and intrinsic motivation [13, 14], its impact on enhancing teachers’ AI literacy remains less explored. This work complements earlier work on need-supportive environments [61], adding that school contexts that respect autonomy, nurture competence, and build relatedness do more than boost motivation, as they also shape how teachers engage with digital innovation. By confirming this mediating pathway, the present study provides additional insight into how schools can foster AI literacy not only through external resources but also by addressing teachers’ internal psychological drivers. This dual pathway approach enriches existing models of teacher technology adoption, offering a more balanced view than models that emphasize only environmental inputs.

In sum, BPNS does not merely accompany school support—it helps explain how and why such support becomes effective in enhancing AI literacy. Recognizing this mediating role deepens our understanding of teacher growth in AI integration, both practically and theoretically.

### Need-supportive strategies and the development of teachers’ AI literacy

The qualitative findings underscore the pivotal role of school support strategies in addressing university English teachers’ basic psychological needs and, consequently, fostering their AI literacy. Teachers’ expectations for AI integration reveal both the shortcomings of current school initiatives and the concrete pedagogical challenges they face. This study identifies three core dimensions of support: autonomy, competence, and relatedness, each corresponding to teachers’ needs for self-directed learning, technical mastery, and professional connectedness in the process of AI integration.

In terms of autonomy support, teachers highlighted the importance of strategies such as providing opportunities for self-directed learning and offering financial assistance. They sought flexibility in choosing learning approaches, experimenting with AI technologies, and applying it to their teaching practice. These expectations align with the autonomy component of SDT theory, which accentuates volition and self-regulation in one’s learning process. Recent research suggests that AI should be designed to complement rather than constrain, teachers’ decision-making authority, thereby reinforcing their sense of agency [95]. Similarly, Walter’s research on self-directed learning and AI literacy draws attention to the importance of autonomous exploration in teachers’ technological development [96]. In addition, financial assistance was viewed as a critical enabler, allowing teachers to overcome the high costs of AI-related tools and professional training, and thus empowering them to pursue independent, sustained learning.

Regarding competence support, teachers proposed strategies centered on competence enhancement, such as context-specific training, continuous technical support, and personalized feedback. These measures are intended to strengthen professional efficacy through iterative practice and reflection. Consistent with Kimmons and Hall’s findings on effective technology integration [97], teachers need not only conceptual understanding but also ongoing guidance in authentic teaching contexts. Through repeated application and tailored feedback, teachers can refine their pedagogical use of AI, leading to a stronger sense of competence and confidence. This corresponds directly to the competence dimension of SDT, which highlights mastery achieved through successful, meaningful experiences.

For relatedness support, teachers also emphasized the significance of social and relational strategies, including the establishment of learning communities, encouragement of interdisciplinary collaboration, and development of peer mentoring networks. These initiatives reveal that teachers’ adaptation to AI technologies extends beyond technical proficiency. It also depends on their sense of belonging and professional identity within a collaborative culture. Research by Knobel and Kalman [98] reinforces this notion, suggesting that peer learning and social connectedness are central to teachers’ technological adaptation. A supportive community fosters both informational exchange and emotional resilience, ensuring teachers with psychological security to navigate technological transformation.

The support strategies identified in this study illustrate a dynamic interplay between school policy, organizational culture, and the cultivation of teachers’ AI literacy. Addressing teachers’ multifaceted needs requires support that goes beyond technical assistance to include emotional, social, and professional dimensions. Such comprehensive support empowers teachers to make thoughtful, ethical decisions in language education and promotes collaboration to collectively advance AI literacy.

### Theoretical and practical implications

#### Theoretical implications

This study extends Self-Determination Theory (SDT) by showing how school support fosters teachers’ AI literacy through fulfilling autonomy, competence, and relatedness needs. While SDT has traditionally focused on intrinsic motivation, this study links teachers’ BPNS directly to AI literacy development. The findings also offered a novel perspective on school management and teacher development frameworks. Institutional resources, training, and flexibility not only meet psychological needs but also enhance teachers’ confidence and initiative in adopting AI technologies. Moreover, this study enriches technology adoption models by emphasizing that AI literacy depends on both external support and BPNS. School support thus serves as both an external enabler and an intrinsic motivator in developing teachers’ AI literacy.

#### Practical implications

The findings suggest several practical recommendations for school leaders and teacher educators. First, schools should implement tiered, needs-oriented AI training programs covering foundational knowledge, advanced applications, and ethics to meet teachers’ developmental needs. Second, collaborative learning environments—such as peer mentoring, learning communities, and teamwork—should be fostered to support shared AI practices. Third, localized AI literacy standards should be developed with reference to global frameworks, such as UNESCO’s 2024 AI Competency Framework for Teachers, and adapted to local institutional and cultural needs.

Nonetheless, implementation remains challenging, as it requires not only policies or training but also a supportive culture, effective leadership, and sustained collaboration to address teachers’ varied psychological needs in a comprehensive manner.

## Conclusion and future direction

This study, rooted in SDT, utilized a mixed-methods approach to examine the relationship between school support, university English teachers’ BPNS, and AI literacy development. Findings for RQ1 revealed that school support positively influences teachers’ BPNS, with BPNS acting as a key mediator between school support and AI literacy. For RQ2, the study identified several key school support strategies that effectively meet teachers’ BPNS, thereby fostering their AI literacy.

Despite these insights, this study has several limitations. First, there may be a gap between teachers’ self-assessed AI literacy and their actual application skills. Second, although SEM validated the SDT-based school support model’s effectiveness in enhancing AI literacy, implementing these strategies across varied school contexts may be challenging. Additionally, the sample is limited to a specific cultural context, and the applicability of these strategies in different cultural settings warrants further exploration.

Future research could use diverse data collection methods to explore the gap between teachers’ perceived and actual AI literacy and adopt longitudinal designs to examine the long-term impact of AI literacy development. These efforts would provide deeper insights into the process of teachers’ AI literacy growth, offering stronger theoretical foundations and practical guidance for the responsible integration of AI in education.

## Supplementary Material

Supplementary Material 1

## Data Availability

The datasets generated and/or analyzed during the current study are not publicly available due to privacy or ethical restrictions, but they are available from the corresponding author upon reasonable request.

## Abbreviations

AI: Artificial Intelligence
SDT: Self-Determination Theory
