# Supplementary material for: Fueling AI literacy through school support: unveiling the mediating role of basic psychological need satisfaction in Chinese university English teachers
Source: BMC Psychol. 2026 Jan 7;14:167. doi: 10.1186/s40359-025-03949-6 (PMC12870354; doi:10.1186/s40359-025-03949-6)
Supplement: Supplementary file 1 — Supplementary Material 1 [file 40359_2025_3949_MOESM1_ESM.docx]

# Appendices

Appendix A Questionnaire items used

| Constructs | Items | Descriptions | Sources |
| --- | --- | --- | --- |
| **When using AI technology in teaching...** | | | |
| School support | SS1 | My school helps me identify areas where more AI technology training is needed. | Lee et al. (2020)  Chiu et al. (2024) |
|  | SS2 | My school provides suggestions for improving AI skills. |  |
|  | SS3 | My school often offers me opportunities to develop new skills in using AI technology in teaching. |  |
|  | SS4 | My school teaches me how to independently solve problems when using AI technology in teaching. |  |
| Autonomy | AU1 | I feel a sense of choice and freedom in what I am doing. |  |
|  | AU2 | I feel that my decisions reflect my true intentions. | Chen et al. (2015); Chiu (2022) |
|  | AU3 | I feel that my choices express my true self. |  |
|  | AU4 | I feel that I have been doing things that genuinely interest me. |  |
| Competence | COM1 | I am confident in my ability to choose AI technologies that are suitable for classroom use. | Chen et al. (2015); Chiu (2022) |
|  | COM2 | I feel that I am very proficient at using AI technology. |  |
|  | COM3 | I feel confident in effectively integrating AI technology into my teaching content. |  |
|  | COM4 | I feel capable of helping my colleagues use AI technology in teaching. |  |
| Relatedness | RE1 | I feel that there is more interaction and collaboration with my colleagues when we use AI technology together. | Chen et al. (2015); Chiu (2022) |
|  | RE2 | I feel that I have built closer connections with my colleagues and students through the use of AI technology. |  |
|  | RE3 | I feel that I receive support and feedback from colleagues and students when using AI technology. |  |
|  | RE4 | I feel that I am part of a supportive and collaborative teaching community. |  |
|  | | | |
| **AI-literacy** | | | |
| Know and understand AI | KNOW1 | I can distinguish between AI tools and non-AI tools. | Ng et al. (2021；2022);  Carolus et al. (2023) |
|  | KNOW2 | I understand in what ways AI technology can assist my English teaching work. |  |
|  | KNOW3 | I can identify AI technology in products or services. |  |
|  | KNOW4 | I feel confident and comfortable when using AI tools (such as smart language learning software). |  |
|  | KNOW5 | I believe that teachers should actively learn to use AI technology to assist in teaching. |  |
| Use and apply AI | USE1 | I can proficiently use AI tools (such as intelligent essay grading systems) to assist my English teaching. | Ng et al. (2021；2022);  Carolus et al. (2023) |
|  | USE2 | I can easily learn new AI tools (such as online language teaching platforms). |  |
|  | USE3 | I can use AI tools to improve the efficiency and effectiveness of my teaching. |  |
|  | USE4 | I can support and guide students in using AI tools (such as English-speaking practice apps). |  |
|  | USE5 | I can effectively integrate AI technology into my course teaching. |  |
| Assess and create AI | ASSE1 | After using a product or service, I can evaluate the functionality and effectiveness of AI tools or services. | Ng et al. (2021；2022);  Carolus et al. (2023) |
|  | ASSE2 | I can select the most appropriate solution from the various options provided by AI tools or services (e.g., choosing AI tools suitable for different teaching goals). |  |
|  | ASSE3 | I can choose appropriate AI tools or services based on specific educational tasks. |  |
|  | ASSE4 | I can select AI assessment tools that match student learning outcomes. |  |
|  | ASSE5 | I can use AI tools or services to provide feedback on students' learning. |  |
| AI ethics | ETH1 | I always adhere to ethical principles when using AI tools (such as treating all students fairly and transparently). | Ng et al. (2021；2022);  Carolus et al. (2023) |
|  | ETH2 | I remain vigilant about privacy and information security issues when using AI tools. |  |
|  | ETH3 | I stay alert to the misuse of AI tools (such as preventing reliance on AI for cheating). |  |
|  | ETH4 | I always consider ethical and safety issues when applying AI technology. |  |
|  | ETH5 | I can promptly identify ethical and moral violations when applying AI and take appropriate measures. |  |

Appendix B Demographic profile of interview participants (N=23)

| Participant  ID | Gender | Teaching Experience | University Type | Administrative Role | Educational Credentials |
| --- | --- | --- | --- | --- | --- |
| P01  P02 | Female | 5 | First-tier | Teacher | Doctorate |
|  | Female | 8 | Second-tier | Teacher | Master's |
| P03  P04  P05  P06  P07  P08  P09  P10 | Female | 15 | Third-tier | Teacher | Master's |
|  | Male | 10 | Vocational | Teacher | Master's |
|  | Female | 20 | First-tier | D Head | Doctorate |
|  | Female | 12 | Second-tier | Teacher | Doctorate |
|  | Male  Female  Female  Male | 7  18  22  6 | Third-tier  First-tier  First-tier  Second-tier | Teacher  D Head  D Head  Teacher | Master's  Doctorate  Doctorate  Master's |
| P11  P12  P13  P14  P15  P16  P17  P18  P19  P20  A01  A02  A03 | Female | 15 | vocational | Teacher | Master's |
|  | Female | 25 | Third-tier | Teacher | Master's |
|  | Female  Male  Female  Female  Female  Female  Female  Female  Male  Male  Male | 4  9  20  11  16  13  17  10  15  20  12 | First-tier  Second-tier  Vocational  First-tier  First-tier  Second-tier  First-tier  Third-tier  First-tier  Second-tier  First-tier | Teacher  Teacher  D Head  Teacher  D Head  Teacher  Teacher  Teacher  V Principal  Dean  Head of Info | Doctorate  Master's  Doctorate  Doctorate  Doctorate  Doctorate  Doctorate  Master's  Doctorate  Doctorate  Doctorate |

"First-tier" = top universities; "Second-tier" = mid-tier universities; "Third-tier" = lower-tier or private universities; "Vocational" = institutions focusing on practical skills. "D Head" = department head; "V Principal" = vice principal; "Head of Info" = head of information management center.

Appendix C Interview Protocol

The following protocol was used to guide the semi-structured interviews with teachers and administrators. The questions were designed to explore participants' experiences and perceptions of school support in relation to AI literacy development. Minor adjustments were made during interviews based on participants' roles and responses.

**Part 1: Teacher Interview Questions**

Q1: To what extent does the school allow you to make autonomous decisions about how and when to integrate AI tools into your teaching? Could you describe any experiences where you felt supported or constrained?

Q2: Do the school's support measures (e.g., training, resources) help you build confidence in using AI tools in teaching? Please provide specific examples.

Q3: Which types of school support have helped you improve your AI-related skills (e.g., technical training, pedagogical strategies, peer support)?

Q4: What major challenges have you encountered in using AI technologies? In your view, has the school provided adequate assistance to overcome them?

Q5: Has the school promoted collaboration and communication among teachers about AI technologies? Can you describe any collaborative experiences?

Q6: From your perspective, what are the school's most effective practices in supporting AI integration? What areas still require improvement?

Q7: Are there any types of support you need but the school has not yet provided?

Q8: If formal school support were unavailable, would you still choose to use AI in your teaching? Why or why not?

**Part 2: Administrator Interview Questions**

Q1: To what extent does the school allow teachers autonomy in selecting AI tools and deciding how to integrate them into their teaching? As an administrator, how do you ensure and support this autonomy in practice?

Q2: To what extent do the school's support measures (e.g., training, resources) enhance teachers' confidence and competence in using AI tools? Please describe your role and experiences in providing these supports.

Q3: Which school support measures have been most effective in enhancing teachers' AI-related skills? How does your team evaluate the effectiveness of these measures?

Q4: What are the key challenges the school has faced in promoting teachers' use of AI technologies?

Q5: Has the school established any platforms to facilitate collaboration on the use of AI in teaching? How have you promoted such collaborative practices?

Q6: From a managerial perspective, what have been the most effective school practices in supporting AI integration? What aspects still require improvement?

Q7: In your opinion, what additional support could the school provide to better assist teachers in using AI in teaching?

Q8: If formal school support were unavailable, do you think teachers would still be motivated to adopt AI technologies? How would you promote their autonomous engagement in such cases?
